# Child Maltreatment and the Mediating Effects of Bullying Victimization on School Engagement Among South Korean Youth in Orphanages and a National Sample

**DOI:** 10.3390/children12070829

**Published:** 2025-06-23

**Authors:** Sunghwan Cho, Seon Kim, Hollee A. McGinnis, Traci L. Wike

**Affiliations:** 1 Department of Social Welfare, The Catholic University of Korea, 43 Jibong-ro, Wonmi-gu, Bucheon-si 14662, Gyeonggi-do, Republic of Korea; scho9595@catholic.ac.kr; 2 Department of Social Welfare, Sunchon National University, Jungang-ro 255, Suncheon-si 57922, Jeollanam-do, Republic of Korea; 3 School of Social Work, Virginia Commonwealth University, 1000 Floyd Avenue, Richmond, VA 23284-2027, USA; hamcginnis@vcu.edu (H.A.M.); twike@vcu.edu (T.L.W.)

**Keywords:** bullying victimization, child maltreatment, orphanage care, school engagement, Republic of Korea

## Abstract

**Background:** Peer bullying is a global problem affecting youth around the world that can impact youth development including school engagement. The relationship between child maltreatment and school bullying victimization and perpetration is well known. However, few studies have explored the extent of bullying among vulnerable groups of adolescents. Youth in orphanage care may be at higher risk of both maltreatment and bullying because of the circumstances and stigma that brought them into care. This study aimed to examine peer bullying victimization among adolescents residing in orphanage care compared to a national sample of adolescents in South Korea, and to investigate the mediating effect of bullying victimization on the relationship between child maltreatment and school engagement. This comparison allowed for an examination of how caregiving contexts may differentially influence the impact of maltreatment and bullying on school engagement. **Methods:** Data from the national Korean Welfare Panel Study (KoWePS) 7th wave (*n* = 521) and a study of 153 South Korean adolescents in orphanage care were merged into one dataset (*n* = 675). Analysis included descriptive and bivariate statistics, two simple mediation analyses, and multiple regression analysis to verify mediation effects using PROCESS Macro. **Results:** Adolescents in orphanage care reported significantly higher bullying victimization and lower school engagement than the national sample. Negative correlations were identified between child maltreatment, bullying victimization, and school engagement among adolescents in orphanage care. Importantly, bullying victimization significantly mediated the relationship between child maltreatment and school engagement within this group. **Conclusions:** These findings highlight the complex relationship between maltreatment and bullying victimization, emphasizing the need for comprehensive interventions addressing both maltreatment and peer bullying to enhance school engagement among vulnerable youth.

## 1. Introduction

Bullying is a common form of youth violence that affects adolescents worldwide. It is generally defined as intentional, repeated aggressive behavior that occurs within the context of a real or perceived power imbalance between the perpetrator and the victim [1]. Recent studies indicate that bullying includes physical, verbal, relational, and cyber forms, and emerges from interactions between individual, peer, school, and societal factors [1,2,3]. Involvement in bullying impacts youth social development and can lead to negative outcomes, including problems with school adjustment, academic success, and peer relationships [4,5], as well as psychosocial problems such as low self-esteem, depression, anxiety, and suicidality [1,6,7].

In addition to these widely documented effects, bullying victimization during adolescence has also been associated with body image disturbances, such as body dissatisfaction and imaginal disorders [8]. Furthermore, the psychological consequences of bullying can be conceptually grouped into internalizing symptoms (e.g., depression, anxiety, somatization) and externalizing symptoms (e.g., aggression, delinquency, substance), which together provide a broad framework for understanding adolescent mental health [9]. For some, the effects of bullying continue well into adulthood, impacting interpersonal relationships and social adjustment [10]. Therefore, addressing bullying violence is a critical issue for youth development and ultimately healthy and productive adulthood [11].

As a global problem, bullying violence affects youth in many cultural contexts [12]; additionally, national comparative research has found that while prevalence rates differ across countries, the consequences of bullying involvement are similar [13]. In South Korea, bullying has received increased attention due to several incidents of youth suicide where bullying was a precipitating factor [13]. Among South Korean youth, prevalence rates of bullying are high, ranging from approximately 10% to 60% of youth self-reported involvement with school bullying as a bully, a victim, or both [13,14]. Studies of youth bullying in South Korea have also identified prevalence rates to be highest in the upper elementary and middle school years, tapering off in high school [13,15].

Several factors have been identified that might increase a youth’s exposure to bullying involvement, including stressful family environments and maltreatment [16]. Within the family context, adverse childhood experiences, including abuse, neglect, and conflict within the home, have been found to place children at risk for peer victimization at school [17,18,19]. Based on social learning theory, experiencing abusive, neglectful, or coercive parenting impacts a child’s perception of healthy interpersonal relationships, which may limit their ability to form positive relationships with peers [20,21]. This, in turn, puts a child at risk of experiencing bullying victimization [22]. In a meta-analysis synthesizing over 70 studies of parenting behavior and its relationship to bullying experiences, maladaptive parenting was found to be positively related to bullying victimization [23].

Among several factors associated with bullying victimization, child maltreatment has been consistently associated with heightened risk [16,17]. Such maltreatment experiences may undermine children’s development of social skills, which are crucial for healthy peer interactions. Deficits in social competence have been observed among children with histories of trauma or institutional care, potentially increasing their susceptibility to bullying victimization [24,25]. This suggests that social skill impairment may be one mechanism through which early adversity contributes to later peer-related difficulties. Maltreatment experiences, including physical and emotional abuse and neglect, may impair children’s ability to develop healthy interpersonal relationships, increasing their susceptibility to peer victimization at school [20,21].

Research on youth growing up in alternative care settings, specifically orphanages (also referred to as institutional care), and bullying victimization is limited. Youth in orphanages may be at greater risk of being bullied given the added burden of social stigma related to orphanage care, institutional deprivation, and exposure to adverse childhood experiences and family risk factors noted above. Although the stigma of being in institutional care has not been researched extensively, one study of adolescents in institutional care in Turkey who experienced more stigma because they lived in an orphanage had higher total emotional and behavioral problems [26]. As noted, the increased vulnerability of youth in orphanages may also be attributed to family-related risk factors that often precede institutionalization and institutional deprivation while in care (for a review, see [27]).

Caregiver maltreatment, abandonment, or neglect is known to disrupt early attachment formation and emotional regulation. These early adverse experiences may compromise children’s ability to build trusting peer relationships and increase their susceptibility to bullying [14]. In addition, while orphanage facilities in South Korea are generally well maintained and provide adequate physical care, they often experience high staff turnover, which may hinder the development of consistent and stable caregiver relationships [28]. Although new caregivers may provide high-quality care, frequent changes can disrupt the continuity of attachment and emotional security. These relational instabilities may result in difficulties with emotional regulation and trust, making children more vulnerable to social problems, including peer rejection and bullying victimization [14].

In South Korea, approximately 24,000 children live in out-of-home care, including group homes, institutional facilities, and foster care settings, according to national child welfare statistics [29]. Among these, over 13,000 children reside in institutional care facilities, commonly referred to as “orphanages”, despite many of them having living biological parents. These children often enter care due to family disruption, abuse, neglect, or economic hardship or abuse, factors which independently increase vulnerability to maltreatment and possible bullying victimization [28]. Given the scale and potential vulnerability of this population, understanding their psychosocial risks—including peer bullying victimization—is a critical public health concern.

Given the established relationship between adverse childhood experiences and bullying involvement, it is important to understand whether adolescents in orphanage care may be more likely to experience bullying victimization than their peers. Emerging research has found higher rates of peer bullying compared to rates in the general school population among South Korean elementary school-aged children who used child welfare facilities (orphanages, group homes, community child centers). In one study, the rate of peer bullying among younger children (ages 6 to 9) was 22%, and for older children (ages 10 to 12), this was 12% [30]. In another study utilizing data from the Panel Study of Korean Children in Out-of-Home Care (PSKCOC), among pre-teens (ages 11 and 12), there were differences in rates of bullying victimization by child care type, with those in group homes experiencing the most, followed by youth in orphanage care, and the lowest among those in foster care [31]. Less research has focused on adolescents (12 and older) in orphanage care and their experiences of bullying victimization.

Previous studies indicate that bullying victimization negatively impacts school-related outcomes, particularly school engagement. Research has also identified a number of school-related factors associated with an increased risk of bullying victimization and lower school outcomes, including negative school climate and student disconnectedness [32,33]. Research consistently shows that bullying victimization correlates with reduced school engagement and poorer academic achievement among South Korean adolescents [34,35,36]. Furthermore, research in South Korea has also found that factors important for academic achievement are lower among youth in orphanage care. For example, studies of younger children in orphanage facilities found they reported significantly lower school life satisfaction [37], as well as more difficulties with school engagement and peer relationships than those raised in healthy biological families [38]. For children in orphanage care, these school outcomes may be further compromised by pre-existing vulnerabilities related to maltreatment, insecure attachments, and inconsistent caregiving environments as noted earlier [38,39]. Hence, youth residing in institutional settings, such as orphanages, may represent a particularly vulnerable group regarding bullying victimization. Despite these risks, limited research has focused specifically on adolescents in orphanage care and their bullying experiences compared to their peers in the general population.

Given the identified gaps in existing research, particularly regarding adolescents in orphanage care, this study aimed to explore the potential mediating effect of bullying victimization on the relationship between child maltreatment on school engagement among South Korean adolescents residing in orphanages compared to a national representative sample. Drawing upon social learning theory, we examined whether patterns of early adversity and peer interaction contributed to educational engagement. Specifically, this study addressed the following research questions: (1) Do adolescents in orphanage care experience higher levels of bullying victimization compared to their peers? (2) Is child maltreatment associated with bullying victimization and school engagement? (3) Does bullying victimization mediate the relationship between child maltreatment and school engagement?

Utilizing social learning theory as a guiding framework, this study posits that children exposed to maltreatment may internalize or imitate aggressive and maladaptive interpersonal behaviors modeled by caregivers or peers. A main assumption of the theory is that behaviors are learned through observation and reinforcement within social environments [40]. In institutional care settings, inconsistent caregiving or exposure to coercive dynamics may reinforce such maladaptive behavioral patterns. These learned behaviors can increase vulnerability to peer bullying victimization and inhibit the development of prosocial engagement in academic settings. Thus, we hypothesized that adolescents in orphanage care would exhibit higher rates of bullying victimization, and that greater bullying victimization would significantly mediate the relationship between childhood maltreatment and school engagement. This conceptualization aligns with social learning theory’s emphasis on the environmental conditioning of behavior through social learning processes and helps clarify the mechanisms associating adverse caregiving contexts with educational disengagement.

## 2. Methods

### 2.1. Participants

Data for this cross-sectional study were obtained by merging the 7th wave (*n* = 521) of the Korean Welfare Panel Study (KoWePS) with a study of 170 Korean youth living in institutional care [41]. KoWePS is a national longitudinal panel study that began in 2006 and was conducted by the Korea Institute for Health and Social Affairs and Seoul National University [42]. Data are collected annually on households (parent report) with an oversampling of low-income families, relating to their welfare status, economic conditions, and social security, with additional surveys conducted with people with disability and youth in various years. In initial wave 1, conducted in 2006, a national random sample of 7027 households were surveyed, including surveys administered directly to the children of each household (*n* = 759). Self-reported child data were also collected in wave 4 in 2009 (*n* = 609) and in wave 7 in 2012 (*n* = 521), with the attrition of the total number of households falling by 74.53% from the first wave.

Wave 7 of KoWePS was chosen for this study because data were collected around the same time as the data of 170 adolescents residing in 10 institutions in the Seoul Metropolitan Area and a southern province collected between March 2014 and January 2015. The convenience sample of adolescents in orphanage care was collected as part of another cross-sectional study examining school and mental health problems that included maltreatment, behavioral problems, and school measures from the KoWePS self-report surveys of youth in households [41]. Furthermore, KoWePS Wave 7 youth were similar in age to the adolescents in orphanage care, with most being high school students (10th to 12th graders in the U.S.), including youth who had dropped out or were on a leave of absence from school.

Adolescents in orphanage care who were younger than 12 years old were excluded from the current study, so the ages were more similar in both datasets. Data from Wave 7 of the KoWePS supplementary youth survey (*n* = 521) were merged with the adolescents in orphanage care (*n* = 154) to create a new dataset of youth (*n* = 675) that were analyzed for the present study.

### 2.2. Measures

School Engagement. The measure of students’ perceived psycho-social engagement in school consisted of nine items: school being fun, liking to learn most subjects, respect for most teachers, having a good attitude, regularly doing homework, following teacher instructions, attempts to quit school, cheating on an exam, and leaving class without permission. The 3-point Likert responses ranged from 0 (not at all) to 3 (always). The measure was summed, with higher scores indicating more school engagement. The Cronbach’s alpha was 0.75 and was considered to have reliable internal consistency [43].

Bully Victimization.This peer victimization scale consisted of six items: verbal aggression (being teased or verbally taunted), relational aggression (intentionally being excluded; spreading bad rumors), intimidation (for not doing what another student wanted them to do; being intimidated or scared for money or property), and physical aggression (being hit, kicked, or punched by other children). All responses were on a 4-point Likert scale ranging from not at all (0) to always (3). All items were summed so that higher scores indicated more frequent bullying victimization. The coefficient of internal consistency was 0.671. While this is within a moderate range, it is slightly below the recommended threshold of 0.70 for acceptable reliability [44]. Due to the limited number of items per bullying subtype, we were unable to perform factor analysis or assess each form of victimization separately.

Child Maltreatment. This variable assessed the frequency of maltreatment by a caregiver or parent in the past year. The scale consisted of 8 items capturing physical abuse (i.e., “I have been hit badly by my caregiver”), emotional abuse (i.e., “My parents made me feel shame and humiliation”), and neglect (i.e., “My parents notice if I need things”). Responses were on a 5-point Likert scale ranging from never (0) to once or twice per year (1), once or twice per 2–3 months (2), once or twice per month (3), and about once or twice per week (4). Cronbach’s alpha was excellent at 0.773 [44]. The measure was summed so that higher scores indicated more frequent exposure to child maltreatment over the past year.

Control Variables.Three variables were controlled for those that were likely to influence the relationship between bullying victimization and school engagement: age, gender, academic performance (mean score across all subjects), and orphanage care or national sample (KoWePS). Age was treated as a continuous variable calculated by the student’s date of birth and the time they participated in this study. Gender was treated dichotomously as male (0) and female (1). A dichotomous variable was created for participants in the national dataset (0 = national) and the orphanage group (1 = orphanage) to compare youth in orphanage care and the national sample.

### 2.3. Analytic Methods

Stata 17.0 MP and Software Package for Social Science (SPSS) 27.0 were used for data analysis. The KoWeps dataset was limited to variables of interest that matched the orphanage group dataset (school engagement, maltreatment, and bullying victimization), and the orphanage dataset was limited to youth aged 12 and older. Then, the two datasets were merged together to create one dataset (*n* = 675). After merging the datasets, the final dataset was checked for duplicate data and formatted.

Prior to the main analyses, we conducted chi-square tests and *t*-tests to examine baseline group differences across key demographic variables, including gender (χ^2^ = 15.03, *p* < 0.001), school grade (χ^2^ = 338.65, *p* < 0.001), and age (*t* = 13.07, *p* < 0.001). These preliminary analyses revealed statistically significant differences between the two groups in all three variables.

Univariate statistics were run to describe the demographics and characteristics of this study. Next, bivariate statistics were used to identify the association between the variables of interest. A simple mediation analysis was conducted using PROCESS Macro. Multiple regression analysis was run to verify the mediation effects of child maltreatment between bullying victimization and school engagement, controlling for demographic variables and group. To test the mediation effect, 5000 bootstrap resamples were conducted.

## 3. Results

### 3.1. Demographic Characteristics

The sample from the national KoWeps dataset comprised 521 adolescents, ranging from 12 to 19 years old (*M* = 16.86 years, *SD* = 0.89) (Table 1). Participants were 270 males (51.8%) and 251 females (48.2%). Most youth were attending high school (95.2%), and the percentage of school dropouts and leave of absence (4.8%) was low. Nearly the full KoWeps sample (94%) were living with family.

The sample size of adolescents in orphanage care in this study was 154 youth, ranging in age from 12 to 19 years old (*M =* 15.03 years, *SD* = 1.86). Of these youth, 107 were male (69.5%) and 47 were female (30.5%). In addition, 57.1% were in middle school and 42.9% were in high school. The dominant reason for placement in the orphanage was parental absence or inability (70.2%) and marital problems (29.8%). Excluding non-responses, the average academic performance mean scores were 3.01 (*SD* = 0.97) for the KoWeps group (*n* = 496) and 2.44 (*SD* = 1.13) for the orphanage group (*n* = 154).

### 3.2. Group Differences: Maltreatment, Bullying Victimization, and School Engagement

Results of the *t*-test analysis for between-group differences on all the variables in this study are presented in Table 2. The orphanage group had statistically significantly higher mean scores on school bullying victimization (*t* = −3.589, *p* < 0.001) than the KoWePS group. Otherwise, the mean score on school engagement (*t* = 2.907, *p* = 0.004) was statistically higher in the KoWePS group than the orphanage group.

Table 3 presents the results of the *Pearson correlation* analysis among all study variables. Higher levels of school bullying victimization were significantly correlated with lower school engagement (*r* = −0.240, *p* < 0.01), higher maltreatment (*r* = 0.283, *p* < 0.01), younger age (*r* = −0.112, *p* < 0.01), male gender (*r* = −0.093, *p* < 0.05), belonging to the orphanage group (*r* = 0.184, *p* < 0.01), and lower academic achievement (*r* = −0.140, *p* < 0.01).

Higher school engagement was significantly correlated with belonging to the national sample group (*r* = −0.127, *p* < 0.01), higher academic achievement (*r* = 0.457, *p* < 0.01), lower school bullying victimization (*r* = −0.240, *p* < 0.01), and lower maltreatment (*r* = −0.186, *p* < 0.01).

### 3.3. Mediating Effect of Bullying Victimization on the Relationship Between School Child Maltreatment and School Engagement

Multiple regression models were conducted to test the mediating effect of school bullying victimization (Table 4). The results of the mediated multiple regression analysis provide strong evidence for a significant indirect pathway linking child maltreatment to school engagement through school bullying victimization. In Model 1, child maltreatment was significantly positively associated with higher levels of school bullying victimization (B = 0.156, *p* < 0.001). Additionally, youth residing in orphanages reported significantly more bullying experiences compared to those in the general population (B = 0.586, *p* < 0.01). In Model 2, school bullying victimization was shown to be significantly negatively associated with school engagement (B = −0.326, *p* < 0.001), and child maltreatment also had a significant direct negative effect on school engagement (B = −0.143, *p* < 0.01). Furthermore, grade level was positively associated with school engagement (B = 1.491, *p* < 0.001). In Model 3, when school bullying victimization was excluded, the effect of child maltreatment on school engagement became stronger (B = −0.194, *p* < 0.001), suggesting that bullying partially mediates the relationship between maltreatment and engagement.

Child maltreatment had a significant total effect on school engagement (B = −0.194, *SE* = 0.043, *t* = −4.483, *p* < 0.001). When peer bullying victimization was included as a mediator, the direct effect of child maltreatment on school engagement remained significant (B = −0.143, *SE* = 0.045). The indirect effect through bullying was also statistically significant (B = −0.050, *SE* = 0.018), with a 95% bootstrap confidence interval that did not include zero [−0.090, −0.019], suggesting a statistically significant indirect association. These findings suggest that bullying victimization partially mediates the relationship between child maltreatment and school engagement, consistent with theoretical expectations.

## 4. Discussion

This study aimed to extend knowledge on school peer bullying victimization among a vulnerable population of youth, adolescents residing in orphanage care, and to compare their experiences to a national sample of adolescents in South Korea. In addition, this study sought to contribute to the understanding of how risk factors, such as bullying victimization and maltreatment, are related to important protective factors in schools (i.e., school engagement) that ultimately contribute to educational achievement and well-being.

Our hypothesis, stating that adolescents in orphanage care would experience greater bullying victimization compared to the general adolescent population, was confirmed. The adolescents in orphanage care in this study were all attending schools in the communities in which the facility was located. Based on prior research and cumulative risk theory, it was thought that maltreatment, in addition to neglect, may place adolescents in orphanages at greater risk for peer bullying victimization. Children with maltreatment histories often experience difficulties managing emotions and behaviors, potentially increasing their vulnerability to peer victimization [45]. Maltreatment-related emotional dysregulation and difficulties in interpersonal relationships may exacerbate negative peer interactions and ultimately decrease school engagement [46,47].

It may be that the statistically higher mean scores on peer bullying victimization were partly because of age differences between the samples. In the national sample, almost the entire group of adolescents were in high school, whereas in the sample of youth in orphanage care, 57.1% were in middle school and 42.9% were in high school. Other studies of school bullying victimization in South Korea have found age differences in the incidence of school bullying victimization, with rates higher in middle school than high school. For instance, in one study, the rates were highest among youth in the 6th grade of primary school and the 1st grade of middle school, equivalent to the U.S. educational system for middle school grades 6th and 7th [48]. This was consistent with findings in another study of 2936 South Korean students by [13] that found that the prevalence rates of bullying victimization by age group were highest among students in the upper elementary school levels (ages 10–12), followed by middle school (ages 13–14; 8.3%) and high school (ages 15–17; 6.4%). Nonetheless, the current study provides support for the fact that while bullying victimization may decrease in high school, adolescents in orphanages are at risk for experiencing more peer bullying victimization.

It could also be that adolescents in orphanage care are more vulnerable to being victims of peer bullying because of their living situation in an orphanage. Adolescents in orphanages in the current study did not differ from the national sample on the average exposure to maltreatment over the past year, suggesting the relatively high quality of institutional care in South Korea. Nevertheless, the institutional nature of care and pre-institutional adversities may still be factors. Furthermore, social stigma could be another factor. One culturally specific form of bullying in South Korea is called *wang-tta*, meaning a person targeted for being bullied [28]. In one qualitative study of adolescents in South Korean orphanages, youth reported that they struggled to reveal to their peers their status of living in an orphanage out of fear of being ridiculed and becoming a *wang-tta* [49]. Future research needs to include measures of social stigma associated with living in an orphanage, or other differences related to family formation, that may be additional risk factors for school bullying victimization for certain groups of youth.

Adolescents in orphanage care in the present study also had average scores that were statistically lower on school engagement. This finding is consistent with prior research of younger children in orphanage facilities in South Korea [36,37] and extends this finding to adolescents in institutional care. This finding may be explained by attachment theory [50,51,52]. Children who experience relational trauma and disruptions from attachment figures are more likely to develop insecure styles of attachment. This can include indiscriminate attachment disorders in which a child may lack appropriate social boundaries or be overly friendly with strangers, which may be distorted perceptions of social support (i.e., perceiving there to be more social support than there is). Insecure attachment has been found to be associated with externalizing behavior problems (see meta-analysis by [53] Van IJzendoorn, Schuengel, & Bakermans-Kranenburg, 1999), as well as difficulty in regulating emotions, including affective connections that may affect school engagement.

Study findings on the negative correlation between maltreatment, bullying victimization, and school engagement align with previous research, indicating that negative social experiences in the family and in the school environment are related to students’ engagement in school [7,36]. The negative correlation between bullying victimization and school engagement highlights the importance of fostering a safe and supportive school environment. Consistent with previous studies, students who experience maltreatment and bullying may exhibit reduced enthusiasm for school life, which can adversely affect their overall academic achievement [31] and their level of school engagement [7,36]. Furthermore, child maltreatment may directly exacerbate experiences of bullying victimization, compounding their effects on students’ ability to actively engage in educational settings. This layered vulnerability reflects a cumulative disadvantage, where not only are children psychologically impacted by interpersonal adversity, but their capacity to benefit from educational opportunities is also concurrently impaired [54].

The present study also aimed to elucidate the mediating role of bullying victimization in the relationship between child maltreatment and school engagement. The mediation analyses conducted confirmed our hypothesis, demonstrating that bullying victimization significantly mediates the relationship between the experience of child maltreatment and the level of engagement in school activities. These findings highlight statistical associations between bullying victimization experiences and the broader psychosocial environment of the children. Specifically, it was observed that the negative consequences of bullying victimization extend beyond immediate psychological distress, permeating into the children’s relationships with their engagement with the school environment. The mediation analyses demonstrated statistically significant associations among bullying victimization, child maltreatment, and school engagement. However, due to the cross-sectional nature of the data, these findings should be interpreted as statistical associations consistent with the proposed theoretical model, rather than as evidence of a causal or temporal mediation pathway.

Moreover, the inclusion of age, gender, academic performance, and orphanage group status as variables in models 1 and 2 of the multiple regression analyses underscores the importance of considering demographic factors and life circumstances in understanding the experiences of maltreated and bullied children. The compounded negative effects of these demographic factors highlight the need for targeted interventions that address the unique vulnerabilities of these populations.

Finally, while the data used in this study were collected prior to the COVID-19 pandemic, they still offer meaningful insights. In particular, the data provide a valuable pre-pandemic baseline for understanding the mechanisms and empirical evidence linking child maltreatment, bullying victimization, and school engagement. Given that child-reported, nationally representative datasets in South Korea remain rare, especially those including vulnerable subgroups such as institutionalized youth, this study contributes to the field by leveraging unique and underutilized data sources. Although more recent studies are needed to assess post-pandemic changes in school bullying dynamics, the theoretical relationships examined here remain relevant across temporal contexts.

### 4.1. Implications for Practice and Policy

Social workers, educators, and policymakers should utilize the findings of this study to support and promote policies that acknowledge and address the complexities of bullying victimization and child maltreatment, and the need to attend to vulnerable populations of youth, such as those who are in alternative care settings like orphanage care. This entails not only establishing policies to protect children from such encounters but also comprehensive practices that address the far-reaching consequences of bullying on children’s educational experiences and their wider social interactions.

The efficacy of multifaceted interventions aimed at mitigating school violence remains inadequately assessed, despite the concerted efforts of governmental entities, non-governmental organizations, community stakeholders, and educational institutions in South Korea. These initiatives, including the implementation of legislative frameworks for the prevention and management of school violence, have attempted to incorporate pedagogical perspectives, victim–offender considerations, and the idiosyncratic nature of school-based aggression. However, the practical application and enforcement of these measures have not yielded demonstrably positive outcomes, calling into question their overall effectiveness in addressing this complex social issue [55].

Thus, there is a need to advocate for legislative measures that promote comprehensive anti-bullying initiatives and facilitate the integration of social work services within educational institutions. The development of such policies should be informed by empirical evidence and incorporate insights from practicing social workers, youth with lived experiences, and educators, thereby reflecting the pragmatic realities and exigencies of the field. One suggestion of a comprehensive anti-bullying initiative is that upon the identification of bullying victimization or child maltreatment incidents, a prompt, coordinated, and tailored response should be implemented, leveraging educational, legal, and therapeutic resources to address the unique needs of affected children.

There exists a pressing need for policies that enable the seamless integration of educational and social service systems in South Korea. There is a need for budgetary provisions that not only ensure the presence of social workers in educational settings but also facilitate the establishment of programs offering sustained support to children who have experienced bullying and maltreatment. Additionally, policy considerations should encompass provisions for the ongoing professional development of social workers, ensuring they possess the requisite knowledge and skills to effectively address the evolving challenges of bullying and maltreatment within the school milieu. Through the provision of practical guidance and influence on policy formulation, social workers can make significant contributions towards mitigating the adverse effects of bullying victimization and child abuse on children. These efforts are directed towards fostering a safe, supportive, and engaging educational environment for all students.

### 4.2. Limitations

Although this study had many strengths, it also had several limitations. First, the two samples were collected at different times. The national youth group sample was collected in 2012, while the orphanage group youth sample was collected between 2014 and 2015. Also, these two groups differ in their composition of gender, age, and grade level. Therefore, the interpretation of the study findings should be approached with caution, as these demographic differences may have influenced the observed outcomes. Second, this study used cross-sectional data. Therefore, it was hard to clarify any causal relationship between the variables. Future studies need to consider longitudinal methods to identify any causal relationships. Third, the Cronbach’s alpha for some study measurements was slightly below the acceptable level. In general, over 0.7 has been treated as an acceptable internal consistency; however, measurements of school engagement and bullying victimization had Cronbach’s alphas under 0.7. Future studies need to address these measurement limitations.

Also, given the cross-sectional design, the mediation effects identified in this study are best interpreted as hypothetical and statistically derived rather than as evidence of causal pathways. Lastly, the data were collected prior to the COVID-19 pandemic, and it is possible that school bullying prevalence and dynamics have since shifted due to changes in academic environments and social interactions. This may limit the generalizability of the findings to current post-pandemic contexts.

## 5. Conclusions

This study highlights the complex interactions between child maltreatment, bullying victimization, and school engagement among South Korean youth, especially those in orphanage care. Our findings demonstrate that bullying victimization significantly mediates the negative relationship between child maltreatment and school engagement. This underscores the extensive impact of maltreatment and bullying on students’ educational experiences and relationships. This study also emphasizes the unique vulnerabilities of youth in orphanage care and calls for targeted interventions and integrated policies to address these issues comprehensively. Our research underscores the critical need for safe and supportive school environments to improve the well-being and educational outcomes of all children, but most especially for the most vulnerable of children.

## Figures and Tables

**Table 1:** Demographic characteristics for study sample by group (N = 675).

Demographics	KoWeps Group		Orphanage Group	
	N	%	N	%
Gender				
Male	270	51.8	107	69.5
Female	251	48.2	47	30.5
Age				
12–15	25	4.8	93	60.4
16–19	496	95.2	61	39.6
Education				
Middle School (Grades 5–9)	0	-	88	57.1
High School (Grades 10–12)	496	95.2	66	42.9
Dropout, Leave, Absent	25	4.8	-	-
Living Arrangement				
Living with the family	490	94.0	-	-
Living separately	31	6.0	-	-
Reason for Placement				
Marital Problem	-	-	37	29.8
Parental Absence or Inability	-	-	87	70.2
Grade Mean (*M* ± *SD*)	3.01 ± 0.97 (*n* = 496)	-	2.44 ± 1.13 (*n* = 153)	-
Total	521	100	154	100

Note. Grade Mean was calculated from participants with valid academic records (*n* = 496 in KoWeps group; *n* = 152 in Orphanage group).

**Table 2:** Between-group differences on means of all variables by group.

	KoWePS Group Mean (SD)	Orphanage Group Mean (SD)	t
Bullying Victimization	0.5 (1.3)	1.2 (2.3)	−3.589 ***
Child Maltreatment	1.3 (2.7)	1.3 (3.5)	−0.082
School Engagement	19.3 (3.4)	18.2 (4.2)	2.907 ***

Note. *** *p* < 0.001.

**Table 3:** Pearson correlation across all variables.

	Age	Gender (ref. Female)	Group	Grade	Bullying Victimizaion	Child Maltreatment	School Engagement
Age	1						
Gender (ref. Female)	0.069	1					
Group (ref. Orphanage)	−0.567 **	−0.149 **	1				
Grade	0.142 **	0.033	−0.233 **	1			
Bullying Victimization	−0.112 **	−0.093 *	0.184 **	−0.140 **	1		
Child Maltreatment	−0.009	0.006	0.003	−0.069	0.283 **	1	
School Engagement	0.016	0.045	−0.127 **	0.457 **	−0.240 **	−0.186 **	1

Note. * *p* < 0.05, ** *p* < 0.01.

**Table 4:** Mediated multiple linear regression.

Variable	Model 1 (MV: School Bullying)		Model 2 (DV: School Engagement)		Model 3 (DV: School Engagement)	
	*B*	*SE*	*B*	*SE*	*B*	*SE*
Constant	0.796 ***	0.932	19.205 ***	1.908	18.945 ***	
Age	−0.001	0.054	−0.244 *	0.110	−0.244 *	0.112
Gender (ref. Female)	−0.224	0.122	0.115	0.251	0.188	0.253
Grade	−0.130 *	0.060	1.491 ***	0.123	1.533 ***	0.124
Group (ref. Orphanage)	0.586 **	0.177	−0.394	0.367	−0.585	0.368
Child Maltreatment	0.156 ***	0.021	−0.143 **	0.045	−0.194 ***	0.043
School Bullying	-	-	−0.326 ***	0.081	-	-
Model Summary	*R* ^2^	*F*	*R* ^2^	*F*	*R* ^2^	*F*
	0.125	18.229 ***	0.258	36.799 ***	0.239	39.979 ***
Relationship	Total effect (*SE*)	Direct Effect (*SE*)	Indirect effect (*SE*)	CI		*t*
				LL	UL	
	0.43	0.045	0.018	−0.2786	−0.1089	−4.4831

Note. CI and *t* value of the total effect. * *p* < 0.05, ** *p* < 0.01, *** *p* < 0.001.

## Data Availability

The KoWePS data used in this study are publicly available from the KoWePS website. The other dataset, collected as apart of Hollee A. McGinnis’s dissertation research, contains potentially sensitive personal information. The merged dataset can be shared upon request with the approval of the authors.
